# Re-development of mental health first aid guidelines for supporting Aboriginal and Torres Strait islanders who are experiencing suicidal thoughts and behaviour

**DOI:** 10.1186/s12888-018-1809-5

**Published:** 2018-07-16

**Authors:** Gregory Armstrong, Natalie Ironfield, Claire M. Kelly, Katrina Dart, Kerry Arabena, Kathy Bond, Nicola Reavley, Anthony F. Jorm

**Affiliations:** 1 Nossal Institute for Global Health, Melbourne School of Population and Global Health, University of Melbourne, 333 Exhibition St, Melbourne, VIC 3000 Australia; 2 Centre for Mental Health, Melbourne School of Population and Global Health, The University of Melbourne, 207 Bouverie St, Carlton, VIC 3010 Australia; 3 Mental Health First Aid Australia, Level 6, 369 Royal Parade, Parkville, VIC 3053 Australia; 4 Indigenous Health Equity Unit, Melbourne School of Population and Global Health, University of Melbourne, 207 Bouverie St, Carlton, VIC 3010 Australia

**Keywords:** Suicide, Indigenous, Aboriginal and Torres Strait islander people, Mental health first aid, Prevention, Helping behaviour, Assistance

## Abstract

**Background:**

Suicide is a leading cause of death among Indigenous Australians. Friends, family and frontline workers (for example, teachers, youth workers) are often best positioned to provide initial assistance if someone is suicidal. Culturally appropriate expert consensus guidelines on how to provide mental health first aid to Australian Aboriginal and Torres Strait Islander persons who are experiencing suicidal thoughts or behaviour were developed in 2009. This study describes the re-development of these guidelines to ensure they contain the most current recommended helping actions.

**Methods:**

The Delphi consensus method was used to elicit consensus on potential helping statements to be included in the guidelines. These statements describe helping actions that Indigenous community members and non-Indigenous frontline workers can take, and information they should have, to help someone who is experiencing suicidal thoughts or displaying suicidal behaviour. A panel was formed, comprising 27 Aboriginal and Torres Strait Islander people who have expertise in Indigenous suicide prevention. The panellists were presented with the helping statements via online questionnaires and were encouraged to suggest re-wording of statements and any additional helping statements that were not included in the original questionnaire. Statements were only accepted for inclusion in the guidelines if they were endorsed by ≥90% of panellists as essential or important.

**Results:**

From a total of 301 statements shown to the expert panel, 172 were endorsed as helping statements to be including in the re-developed guidelines.

**Conclusions:**

Aboriginal and Torres Strait Islander suicide prevention experts were able to reach consensus on appropriate strategies for providing mental health first aid to an Aboriginal or Torres Strait Islander person experiencing suicidal thoughts or behaviour. The re-development of the guidelines has resulted in more comprehensive guidance than the earlier version, for which the panel had rated 166 helping statements and had endorsed 52. These re-developed guidelines can be used to inform Indigenous suicide gatekeeper training courses.

**Electronic supplementary material:**

The online version of this article (10.1186/s12888-018-1809-5) contains supplementary material, which is available to authorized users.

## Background

Suicide is a leading cause of mortality for Aboriginal and Torres Strait Islander peoples in Australia, ranking in as the fifth leading cause of death in 2014 [1]. The suicide death rate for Aboriginal and Torres Strait Islander peoples is estimated to be 23.0 per 100,000, which is twice the rate for non-Indigenous Australians [1]. The issue is particularly pronounced among Aboriginal and Torres Strait Islander youth, with a suicide death rate of 52.5 per 100,000 among those aged 15–24, which is approximately four times the rate of their non-Indigenous counterparts [1].

The high rate of Indigenous suicide is a distressing phenomenon that is similarly plaguing several other postcolonial countries, including Canada, the United States and New Zealand [2]. Suicide among Indigenous peoples is a complex socio-cultural, political, biological and psychological phenomenon that needs to be understood in the context of colonisation, loss of land and culture, trans-generational trauma, grief and loss, and racism and discrimination [3–5]. Additionally, the higher levels of social disadvantage experienced by Indigenous peoples increases their exposure to mental disorders, substance abuse and a suite of stressful life events, for example, unemployment, homelessness, incarceration and family breakdown, all of which are well-documented suicide risk factors [6–11].

Despite the high level of need, there is a lack of documented and rigorously evaluated Indigenous suicide prevention programs, and it is evident that there is no one solution [12, 13]. One evidence-based suicide prevention tool is known as ‘gatekeeper training’ [14]. The underlying premise of gatekeeper training is that family, friends and frontline workers (for example, teachers, youth workers) are often best positioned to identify and provide initial assistance to individuals experiencing suicidal thoughts or displaying suicidal behaviour. Gatekeeper training teaches groups of people in the community how to identify and support individuals who are at high risk of suicide and to refer them to appropriate community supports, including mental health services. [15] A systematic review of suicide interventions targeting Indigenous populations in Australia, Canada, New Zealand and the United States found that gatekeeper-training demonstrated encouraging results, with significant short-term increases in participant knowledge, skills, intentions to assist, and confidence in identifying and assisting individuals at risk of suicide [13]. However, there is still limited evidence regarding the maintenance of these changes and there remains a need for future research to examine longer-term outcome measures, for example, referral and treatment patterns and the impact on rates of suicide attempts and deaths. There is also a need for further research to inform the pedagogical approach to developing and delivering Indigenous suicide gatekeeper training programs and how best to harness existing local community knowledge.

‘Mental health first aid’ is defined as the help provided to a person developing a mental health problem, experiencing the worsening of an existing mental health problem or in a mental health crisis, until appropriate professional treatment is received or until the crisis resolves [16]. In 2001, a Mental Health First Aid training program was established in Australia in response to the need for public education about mental illness and its treatment [17]. Later, an Aboriginal and Torres Strait Islander Mental Health First Aid (AMHFA) program was established [18]. The first edition of the AMHFA course was based around a cultural adaptation of the Standard Mental Health First Aid course guided by an Indigenous working group. Subsequently, a second edition was produced based on a series of guideline documents that were developed using Delphi expert consensus studies with Aboriginal or Torres Strait Islander mental health professionals as expert panellists [19]. Based on this series of guideline documents, the AMHFA program sought to provide recommendations as to how to provide initial assistance to an Aboriginal or Torres Strait Islander person with a mental health problem or in a mental health crisis, including depression, psychosis, substance use, or experiencing a traumatic event, a panic attack, suicidal thoughts or engaging in non-suicidal self-injury. AMHFA guidelines were also developed around ‘*Cultural Considerations and Communication Techniques*’ and, later in 2014, around ‘*Communicating with an Aboriginal or Torres Strait Islander Adolescent*’ [19, 20].

The AMHFA program is run through Mental Health First Aid Australia (MHFAA) who use a train-the-instructor style model, whereby they train their pool of accredited AMHFA Instructors, who are Aboriginal or Torres Strait Islander people, in how to deliver the course material to Indigenous community members and non-Indigenous frontline workers in their respective communities, where they are already embedded and have local support. An initial evaluation of the AMHFA program based on roll-out data and qualitative data obtained from focus group discussions found the program to be both culturally appropriate and acceptable to Aboriginal and Torres Strait Islander people [18].

As part of the process outlined above, AMHFA guidelines for assisting an Aboriginal or Torres Strait Islander person experiencing suicidal thoughts or suicidal behaviour were developed in 2009 [19]. The aim of this current study was to use the Delphi methodology to re-develop these guidelines in order to ensure that they reflect current evidence and best practice in suicide prevention, and contain the most current recommended helping actions that can be shared with Indigenous community members and non-Indigenous frontline workers. Further, this study aimed to expand upon the previous guidelines and provide more comprehensive guidance as to how members of the public can provide mental health first aid to an Aboriginal or Torres Strait Islander person experiencing suicidal thoughts or suicidal behaviour. The re-development of these guidelines will help to ensure they are well placed to inform the development of Indigenous suicide prevention gatekeeper training programs developed by MHFAA and others.

## Methods

The Delphi consensus method has been used extensively in health and social research as a method for decision-making processes, including mental health research [21]. The Delphi method provides a platform for obtaining expert consensus on what constitutes best practice in scenarios that cannot be feasibly or ethically subject to a randomised controlled trial. The process involves a series of questionnaires being sent to a group of experts, who do not have to attend group meetings and can respond anonymously. Traditionally, the Delphi method has involved a number of iterations before consensus is achieved. Feedback is given at each stage in order to help experts assess their opinions against those of the group.

We used the Delphi consensus method to elicit consensus on potential helping statements to be included in the guidelines. The development of the guidelines using the Delphi method involved four steps: 1) formation of the expert panel, 2) questionnaire development, 3) data collection and analysis, and 4) guideline development. The same Delphi process was also used to redevelop the mental health first aid guidelines for supporting an Aboriginal or Torres Strait Islander person who is engaging in non-suicidal self-injury, which was also published in this journal [22].

### Panel formation

A panel was recruited, comprising of 27 Aboriginal and Torres Strait Islander people who had expertise in Indigenous suicide prevention through their professional experience. A recruitment advertisement was sent out via the Aboriginal Mental Health First Aid Instructor email list, the Onemda VicHealth Koori Health Unit (University of Melbourne) email list, and the Lowitja Institute email list. The advertisement encouraged people to distribute the flyer across their broader networks. Potential candidates were asked to contact the research coordinator with information on their expertise in Indigenous suicide prevention and were sent a Plain Language Statement prior to participation. The research was approved by the Human Research Ethics Sub-Committee at the University of Melbourne (HREC No.1443056.1). Expert panel members were reimbursed AUD$250 for completing all three survey rounds.

### Questionnaire development

The questionnaire contained statements describing helping actions that Indigenous community members and non-Indigenous frontline workers can take, and information they should have, to help an Aboriginal or Torres Strait Islander person who is experiencing suicidal thoughts or behaviour. Statements were considered acceptable for inclusion in the questionnaire if the working group (comprising the authors) agreed that they described how someone can help a person who is suicidal with clear and non-ambiguous actions.

The statements were sourced from two previous Delphi questionnaires; the first questionnaire was designed to develop the original Aboriginal mental health first aid guidelines for suicidal thoughts and behaviour in 2009 and the second questionnaire was designed to re-develop the mainstream mental health first aid guidelines for suicidal thoughts and behaviour in 2014 [19, 23]. These previous questionnaires were formed through systematic searches of peer-reviewed literature, grey literature, books, websites and online materials, and existing suicide intervention courses, and these literature searches are described in detail elsewhere [19, 23]. The statements in the questionnaire were divided into ten sections based on common themes. The statements derived from the literature were kept as intact as possible to remain faithful to the original wording of the information. Statements were only modified to ensure consistency of format, or where there was concern about the comprehensibility or cultural appropriateness of the information.

### Data collection and analysis

Once panel members had been recruited, they were sent an electronic link to an online questionnaire hosted by SurveyMonkey. Participants responded by rating how important the first aid action statements were to the development of a set of guidelines on providing mental health first aid to an Aboriginal or Torres Strait Islander person who is experiencing suicidal thoughts or displaying suicidal behaviour. Each statement was rated using a five-point scale with the following options: *Essential, Important, Don’t know/It depends, Unimportant, Should not be included*.

Pre-determined criteria were used to assess the outcome for each statement. Statements were immediately included in the guidelines if they were endorsed by ≥90% of panellists as either essential or important. Statements were re-rated in the Round 2 questionnaire if they were rated as essential or important by 80–89.9% of the panel. Statements were immediately excluded from the guidelines if they were rated as essential or important by less than 80.0% of both panel members.

In Round 1, panel members were also invited to make comments on any ambiguity or wording of the statements presented, and to suggest new statements that had not yet been considered, through a feedback textbox at the end of each section of the questionnaire. The working group reviewed all of these comments. Suggestions that contained novel ideas were used to create new helping statements to be included in the subsequent Round 2 questionnaire. Statements that received comments suggesting ambiguity in the interpretation of its meaning were re-phrased to make them clearer and were also included in the Round 2 questionnaire.

The Round 3 questionnaire comprised new statements that were developed from Round 1 feedback and had been presented for the first time in Round 2, but required re-rating in a further round. Statements that still did not achieve consensus after being re-rated were rejected from inclusion in the guidelines.

Following each round of the three rounds, each panellist was sent a report containing a summary of the results from the previous round, with the report personalised to include the individual panellist’s rating for each statement, as well as a table summary of the overall panel’s rating for the statement. This allowed the panellists to compare their rating with the level of endorsement given by the group as a whole and to inform their future ratings for those statements that needed to be re-rated.

### Guideline development

All statements endorsed as either Essential or Important by ≥90% of the panel members were written into a guideline document. One author (NI) drafted the guidelines by writing the list of endorsed statements into sections of prose based on common themes. Where possible, statements were combined and repetition deleted to reduce length. The draft was then presented to the working group, who edited the document to create a set of guidelines that were written in plain English and were easy to follow. A number of drafting iterations were completed before the group agreed upon the final document, a copy of which was sent to each panel member for review. While panellists could not suggest new content at this stage, they were able to provide feedback on the wording and layout of the document to improve clarity and reduce ambiguity.

## Results

### Expert panel members

We recruited 27 expert panel members (19 female, 8 male, age range 28 to 58 years) who completed the Round 1 questionnaire. Of the 27, 92.6% (*n* = 25) were retained in the study, completing the Round 2 and 3 questionnaires. Approximately one-third (37.0%, *n* = 10) of the panel heard about the study through the Onemda VicHealth Koori Health Unit email list, 11.1% (*n* = 3) through the Aboriginal Mental Health First Aid Instructor list, 7.4% (*n* = 2) through a colleague, 3.2% (*n* = 1) through the Lowitja Institute email list, and 40.7% (*n* = 11) were recruited through other pathways, which is unsurprising given that the advertisement encouraged people to distribute the flyer across their broader networks. The panel members came from a range of health and community services roles: 6 panel members were social workers, 6 were Aboriginal Mental Health Workers, 3 were nurses, 2 were GPs, 2 were academics, 2 were Aboriginal Health Workers, 2 were Aboriginal Mental Health Policy Advisors, 1 was an Aboriginal Community Support Worker, 1 was an Indigenous Public Health Officer and 2 were other types of health workers. Many members of the panel also held multiple other community roles (for example, participation in Indigenous suicide prevention evaluations), indicating a high level of community engagement.

Further socio-demographic information on the panel members is provided in Table 1. In summary, the majority of panel members identified as being Aboriginal, with one identifying as Torres Strait Islander and one identifying as both Aboriginal and Torres Strait Islander. There was a broad representation of States and Territories across Australia, with panel members from Victoria, Queensland, Western Australia, New South Wales, Northern Territory, South Australia, Australian Capital Territory and Tasmania. On average, panel members had 11.6 years (range: 1–27 years) of experience in Indigenous suicide prevention. It is important to note that while we recruited a panel of people with professional expertise in suicide prevention, all panel members reported also having had personal experience (outside of their professional role) with suicidal thoughts and behaviour in either themselves, their families, their friends, or in their broader community network. This indicates that panel members were able to draw on both professional and personal experiences when rating the statements in the questionnaires, adding an important richness to their expertise.

**Table 1:** Characteristics of panel members (*n* = 27)

	% (n)
Age group (range:28–58, mean:45.0 years)	
*25–40 years*	22.2% (6)
*41–50 years*	44.4% (12)
*51–60 years*	33.3% (9)
Gender	
*Female*	70.4% (19)
*Male*	29.6 (8)
Indigenous identification	
*Aboriginal*	92.6% (25)
*Torres Strait Islander*	3.7% (1)
*Both Aboriginal and Torres Strait Islander*	3.7% (1)
Years of experience in suicide prevention (mean = 11.6)	
*1–4 years*	7.4% (2)
*5–9 years*	37.0% (10)
*10+ years*	55.6% (15)
State where currently working	
*Victoria*	25.9% (7)
*Queensland*	14.8% (4)
*Western Australia*	11.1% (3)
*New South Wales*	11.1% (3)
*Northern Territory*	7.4% (2)
*South Australia*	7.4% (2)
*Australian Capital Territory*	7.4% (2)
*Tasmania*	3.7% (1)
*Australia wide*	11.1% (3)
Personal (i.e. not professional) experience with suicidal thoughts or behaviour (*n* = 25)	
*In myself*	48.0% (12)
*In my family*	76.0% (12)
*In my friends*	80.0% (20)
*In my broader community network*	84.0% (21)
*No personal experience*	0.0% (0)
*I’d rather not say*	0.0% (0)

### Ratings of the statements

**Fig. 1:**
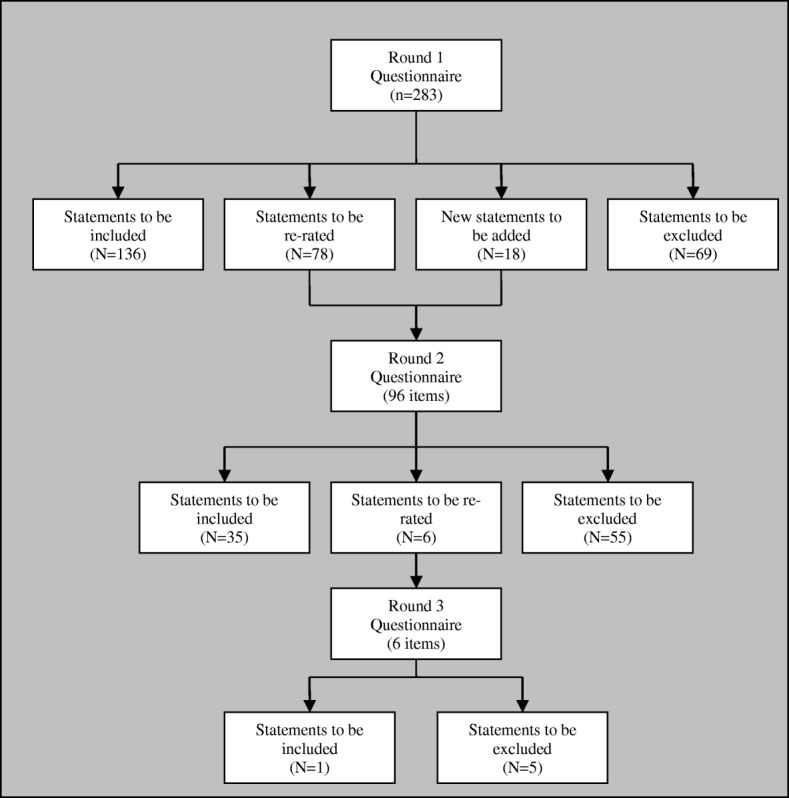
Overview of the three rounds of the Delphi method

An overview of the three rounds of the Delphi study is provided in Fig. 1 and a breakdown of the number of endorsed and rejected statements for each section of the Delphi questionnaire is provided in Table 2. We started with a total of 283 statements in the Round 1 questionnaire, and included an additional 18 new statements based on feedback from the panel, resulting in a total of 301 different statements being rated by the panel across the three rounds. Of these 301 statements, 172 (57%) were endorsed as being either *Important* or *Essential* for the guidelines by ≥90% of panellists; 136 statements were endorsed in Round 1, 35 in Round 2 and 1 in Round 3. A total of 129 (43%) statements were not endorsed for the guidelines; 69 statements were rejected in Round 1, 55 in Round 2 and 5 in Round 3 (see Additional file 1 for a list of all the statements and their respective levels of endorsement).

**Table 2:** Sections in the Delphi questionnaire, and the number of statements endorsed and rejected

Section	Topic	Number of statements endorsed	Number of statements rejected	Total
1	What the first aider should know	27	10	37
2	Identification of suicide risk	29	20	49
3	Assessing seriousness of risk	19	11	30
4	Initial assistance	20	24	44
5	Talking with the suicidal person	39	27	66
6	Safety plan	13	8	21
7	Ensuring safety	1	5	6
8	Passing time during the crisis	1	4	5
9	Confidentiality	8	1	9
10	Adolescent specific	15	19	34
	TOTAL	172	129	301

### Comparison with the original aboriginal mental health first aid guidelines for suicidal thoughts and behaviour

The re-development of the guidelines has resulted in more comprehensive guidance than the earlier version; for the development of the original version, panellists had rated 166 helping statements and had endorsed 52 statements [19]. The re-developed guidelines contain some familiar features while also incorporating some important new guidance for first aiders.

#### Culturally appropriate mental health first aid

The re-developed guidelines reaffirmed some important cultural elements of the original guidelines that are important for people to know when assisting an Aboriginal or Torres Strait Islander person who is having suicidal thoughts. The importance of cultural context and cultural competence was again prominent in the re-developed guidelines, with the endorsement of statements such as the first aider needs “*to be aware that Aboriginal people understand mental health within a wider context of health and well-being, which includes concepts of social and emotional functioning”* and the first aider needs “*to learn about the behaviours that are considered warning signs for suicide in the person’s community, and in doing so take into consideration the spiritual and/or cultural context of the person’s behaviour”*. Cultural safety was also again prominent, for example, with the need for first aiders to be aware of the “*cultural concept of ‘shame’ within the person’s community, and that shame may be triggered by discussing behaviours that may be considered unusual or embarrassing”*; that “*the term ‘help’ may carry negative connotations for some Aboriginal people”*; and that the person has the “*right to make decisions about seeking culturally-based care”*. However, the idea of culturally appropriate first aid was qualified by endorsement of the following statements: “*it is more important to make the person feel comfortable, respected and cared for, than to do all the ‘right things’ and follow all the ‘rules’ when communicating with an Aboriginal person”* and *“it is more important to genuinely want to help than to be of the same age, gender or cultural background”*. The importance of family and community was also prominent, again with a qualification: “*family and friends are a very big part of Aboriginal culture and you should expect involvement by the family and friends in caring for the person. However, you should not assume that all Aboriginal people will want their family involved and respect that the person has the right to choose who they want involved*”. Several statements also noted the need for first aiders to consider that there may be a broad range of potential community supports that may be preferred as sources of support by Aboriginal and Torres Strait Islander people, for example, respected Elders, family and friends, Aboriginal health workers, community liaison officers, youth workers, sports coaches and teachers.

#### Extended guidance in basic communication skills around talking with a suicidal person

An important addition to the re-developed guidelines is the inclusion of extended guidance around the use of basic communication skills in relation to talking with a suicidal person and asking about thoughts of suicide. The re-developed guidelines give more detailed advice around avoiding stigmatising language when asking the suicidal person about suicidal thoughts and specifically addresses an important myth that can stop people from asking about suicidal thoughts: “*If a person is not suicidal, asking them about suicide cannot put the idea in their head. If a person is suicidal, asking them about suicidal thoughts will not increase the risk that they will act on these thoughts, rather, it will allow them the chance to talk about their problems and will show them that somebody cares*”.

Furthermore, while both the original and the re-develop guidelines discuss sourcing professional help, the re-developed guidelines provides additional guidance in basic counselling skills that the first aider can themselves implement when talking with the suicidal person, for example: “*do something to help comfort the person, such as sitting with them, making them a cup of tea, offering them time, friendship and encouragemen*t”; “*allow the suicidal person to do most of the talking*”; and, “*encourage the person to discuss their reasons for dying and their reasons for living, validate that they are considering both options and emphasise that living is an option for them*”. The re-developed guidelines also emphasise new basic communication tips related to ‘listening’ and ‘what not to do’, for example: “*show that you are listening by summarising what the person is saying*”; “*be conscious of your body language, ensuring that it doesn’t communicate a lack of interest or negative attitude*”; “*don’t use guilt or threats to prevent suicide, e.g. do not tell the person they will go to hell or ruin other people’s lives if they die by suicide*”; and, “*don’t give glib reassurance such as ‘don’t worry’, ‘cheer up’, ‘you have everything going for you’ or ‘everything will be alright’*”.

#### Additional considerations when assisting an adolescent who is suicidal

The original guidelines offered no specific guidance for supporting Aboriginal and Torres Strait Islander adolescents. Our recent panel reviewed and endorsed a range of statements that allow the re-developed guidelines to provide additional guidance for when the first aider is supporting an adolescent who is feeling suicidal. These statements appear to be underscored by a major concern about the potential for impulsiveness in youth suicides and the need to closely monitor a suicidal adolescent, while balancing this against the need to involve them in making decisions about the next steps. For example: “*do not leave an adolescent who is feeling suicidal on their own*”; “*make sure someone stays close by them (in the same room, in visual contact) and engage whatever outside resources are available, e.g. family, friend, emergency mental health care or, if necessary, the police*”; “*if the adolescent is reluctant to seek help, you should talk to a helpline or health professional for advice and make sure that someone who is close to the adolescent is aware of the situation*”; and, “*treat the suicidal adolescent with respect and involve them in decisions about who else knows about the suicidal crisis*”.

#### Other areas of difference

Aside from the abovementioned themes, there were a number of other areas where new guidance has emerged in the re-developed guidelines. These include: detail on the use of safety plans; a discussion around times when the first aider may need to breach the confidentiality of the suicidal person; and, a small section on the need for first aiders to look after themselves.

### Comparison with the mainstream (i.E. non-indigenous specific) mental health first aid guidelines for suicidal thoughts and behaviour

There were 217 statements that appeared in both the first round of the current Delphi study and a 2014 Delphi study designed to develop the mainstream (i.e. not specific to Aboriginal and Torres Strait Islander people) mental health first guidelines for suicidal thoughts and behaviour. The 2014 Delphi study had two panels, a consumer panel and a professional panel, whereas the current study only had a professional panel (all of whom also had personal exposure to suicide). The ratings given by our panel of Aboriginal and Torres Strait Islander suicide prevention experts were, on average, statistically significantly higher than those ratings given by the mainstream Delphi panel in 2014. On average, 85.4% of our panel rated each statement as either important or essential for the AMHFA suicide guidelines, compared to an average of 78.1% (t (432) = 4.7, *p* < 0.001) of professionals and 81.1% (t (432) = 3.2, *p* = 0.017) of consumers in the 2014 mainstream Delphi study.

Among those statements rated in both studies, the endorsement ratings given by our Aboriginal and Torres Strait Islander suicide expert panel were strongly correlated across items with both the consumer panel r (221) = 0.79, *p* < 0.05 and the professional panel r (221) = 0.77, *p* < 0.05 from the 2014 mainstream Delphi study. In terms of comparing whether statements were endorsed or not between the two Delphi studies for each of the 223 statements, there was a strong level of inter-rater agreement with a kappa co-efficient of 0.74. In practical terms, the two Delphi studies came to the same conclusion about endorsing or not-endorsing an item on 87.6% of occasions; 92.1% of statements endorsed in the 2014 mainstream Delphi were also endorsed in the current study and 81.3% of statements rejected in the 2014 mainstream Delphi study were also rejected in the current study. The strongly correlated ratings across the two Delphi studies suggest a high degree of overlap in terms of suicide prevention knowledge.

Nevertheless, there were some differences in the ratings among those statements rated in both studies. There were 10 statements endorsed in the 2014 mainstream Delphi that were rejected in the current study and 17 statements that were rejected in the 2014 mainstream Delphi that were endorsed in the current study. Of these 27 statements, there were 16 statements for which our Aboriginal and Torres Strait Islander expert panel had given a markedly different (i.e. ±10%) rating than that given by either the professional or consumer panels in the 2014 mainstream study; 9 statements where there was a marked difference with both the professional and consumer panels, 4 statements where there was a marked difference with the professional panel only, and 3 statements where there was a marked difference with the consumer panel only.

There appeared to be some themes that emerged where there were marked differences with the professional and consumer panels in the 2014 mainstream Delphi study. Firstly, our Aboriginal and Torres Strait Islander panel endorsed items that may reflect the sense of emergency around the issue of suicide in some Aboriginal and Torres Strait Islander communities. For example, our Aboriginal and Torres Strait Islander panel endorsed the following items that were not endorsed in the 2014 mainstream Delphi study: ‘*the first aider should be aware of how commonly suicide occurs*’; ‘*the first aider should not let the person convince them that it is not serious or that they can handle it on their own*’; and, ‘*if the suicidal person won’t make a safety plan, it is not safe to leave them alone for any period of time*’. Secondly, our Aboriginal and Torres Strait Islander panel endorsed statements that may reflect some important cultural communications issues that need to be appreciated by the first aider, with particular emphasis on using a narrative or ‘yarning’ approach so as to avoid asking too many direct questions and taking charge of the situation at the expense of respecting the suicidal person. For example, our Aboriginal and Torres Strait Islander panel endorsed the following items that were not endorsed in the 2014 mainstream Delphi study: ‘*the first aider should begin the conversation by asking the person about how they are feeling’;* ‘*the first aider should keep in mind that asking too many questions can provoke anxiety in the suicidal person*’; and, ‘*the first aider should respect the suicidal person and not try to take charge of the situation*’.

Additionally, there were 33 statements (11.0% of the 301 statements) that were presented to our Aboriginal and Torres Strait Islander expert panel that had not been presented to the earlier 2014 mainstream Delphi panel. These were statements specifically related to cultural competence and cultural safety and new statements that had been derived from the Aboriginal and Torres Strait Islander panel throughout the Delphi study, as well as statements that were similar but had been significantly re-worded to refer to culturally appropriate examples of community supports (for example, respected Elders, Aboriginal Health Workers and community liaison officers) when encouraging the suicidal person to choose someone they would like and would trust to support them. There were another 45 statements (14.6% of the 301 statements) that had been either derived from panellists’ suggestions in the 2014 mainstream Delphi study (and thus had not been rated in the first round of that study) or had been re-worded to the extent that they no longer had exactly the same meaning.

## Discussion

The aim of this study was to re-develop the Aboriginal mental health first aid guidelines for members of the public in providing assistance to an Aboriginal or Torres Strait Islander person experiencing suicidal thoughts or displaying suicidal behaviour. This was achieved by engaging Aboriginal and Torres Strait Islander people who have expertise in the field of Indigenous suicide prevention. Despite being from diverse backgrounds and geographical locations across Australia, the expert panel was able to reach a high level of consensus on a range of mental health first aid techniques, and 172 statements endorsed by ≥90% of panellists were included in the re-developed guidelines.

The re-development of the guidelines has resulted in more comprehensive guidance than the earlier version; our panellists rated 301 statements and endorsed 172, while the previous panel rated 166 helping statements and had endorsed 52 statements [19]. The increase in the number of statements rated by panellists and included in the guidelines is a reflection of the growth of suicide prevention expertise and advice available in the published literature, grey literature, on websites, and other sources. This highlights the importance of conducting revisions of guideline documents, as the advice provided by the literature and expert opinion can change across the span of a few years.

The re-developed guidelines contain some familiar features, while also incorporating some important new guidance for first aiders. They reaffirmed some important cultural elements, under the broad themes of cultural context, cultural competence and cultural safety, as well as reaffirming the importance of family and community when supporting an Aboriginal and Torres Strait Islander person who is experiencing suicidal thoughts. Meanwhile, there were a number of areas where new guidance has emerged in the re-developed guidelines. An important new contribution has been the inclusion of a section on additional considerations when assisting an adolescent who is suicidal. The inclusion of adolescent-specific statements provides recognition that suicidal adolescents may need tailored support, and the statements endorsed by our panel appeared to be underscored by a major concern about the potential impulsiveness of youth suicide [24]. This section carries additional weight given the major concerns around the high rates of suicide among Aboriginal and Torres Strait Islander youth [25], and future re-developments of these guidelines should place greater emphasis on this section as the literature grows in terms of specific advice around Indigenous youth suicide.

The re-developed guidelines also offer extended guidance in basic communication skills around talking with a suicidal person, including communication tips related to ‘listening’ and ‘what not to do’. This guidance is broadly underpinned by the use of a narrative or ‘yarning approach’ that allows the person to do most of the talking and avoids asking too many questions. As Adams, Drew & Walker [26] highlight, when talking about mental health and wellbeing with an Aboriginal or Torres Strait Islander person it is often best to use a narrative or ‘yarning’ approach. Asking too many direct questions or trying to take charge of their situation may make the individual feel ‘shame’, and can result in responses that provide inaccurate information and a sense of disempowerment [26, 27]. Additionally, new guidance has emerged in a section on the use of safety plans, a discussion around times when the first aider may need to breach the confidentiality of the suicidal person, and a small section on the need for first aiders to look after themselves. All of this additional guidance may be especially useful for situations where the first aider is required to be engaged as the primary support for longer periods of time. This is particularly relevant in remote areas where access to immediate ‘professional’ help is not always available or culturally appropriate for Aboriginal and Torres Strait Islander people [28], and situations where the suicidal person is reluctant to talk with others about their suicidal feelings. The extended emphasis on communication tips gives first aiders a greater suite of skills to use when talking to a suicidal person, without detracting from the need to work with the person to identify sources of appropriate help from relevant professionals, family or community leaders.

We observed a high degree of agreement in terms of suicide prevention knowledge between our panel of professional Aboriginal and Torres Strait Islander experts and the non-Indigenous consumer and professional panels who helped construct the mainstream MHFA suicide guidelines [23], in relation to the sub-set of 217 statements that were presented to both the current and former panels. The high proportion of these statements that were endorsed by both panels indicates a moderate degree of transferability of action statements between the mainstream guidelines and the Aboriginal mental health first aid suicide guidelines. We also observed that our panel of professional Aboriginal and Torres Strait Islander experts gave, on average, higher ratings of endorsement for the items compared to the non-Indigenous consumer and professional panels who helped construct the mainstream MHFA suicide guidelines. Future research with Delphi panels comprising Aboriginal and Torres Strait Islander professionals could examine if this is a consistent pattern.

### Important considerations when using the guidelines to support the development of indigenous suicide gatekeeper training programs

The guidelines developed through this study are unique in having been developed using a Delphi methodology to harness the expertise of Indigenous suicide prevention experts from across Australia. The specific purpose of the guidelines is to inform the actions undertaken by mental health first aiders, and the guidelines will be used by MHFAA to revise the curriculum of the AMHFA course to a third edition and to develop a new Indigenous suicide prevention gatekeeper training course to be rolled out by their AMHFA Instructors.

Nonetheless, the guidelines may be useful to others working in Indigenous suicide prevention, particularly those developing or implementing other Indigenous suicide gatekeeper training programs. For example, community members and frontline workers may be hesitant to ask someone directly if they are thinking of suicide for fear that it may put the idea in their head [29]. These guidelines can offer a level of confidence that a panel of Indigenous suicide prevention experts have agreed that it is okay to ask an Aboriginal or Torres Strait Islander person directly if they are having thoughts of suicide. The guidelines make many other important and useful recommendations, for example: taking people seriously when they tell you they are thinking of suicide; providing space to talk about both the person’s reasons for living and their reasons for dying; and not taking charge of the situation for the person but rather encouraging them to make decisions regarding how and by whom they would like to be supported during a crisis, including tapping into important community-based resources like family, friends, Aboriginal health workers, respected Elders and mental health services.

However, it is important to consider some of the following issues before using these guidelines to inform Indigenous suicide gatekeeper training programs. Firstly, the guidelines should not be used in isolation. There are other Aboriginal Mental Health First Aid Guidelines that could also be referred to, most notably the guidelines on ‘*Cultural Considerations and Communication Techniques*’ and ‘*Communicating with an Aboriginal or Torres Strait Islander Adolescent*’ [19, 20]. Those implementing the recommendations as first aiders will also need other local knowledge relevant to their respective communities. Aboriginal and Torres Strait Islander communities are not homogenous and, as such, reading these generalised guidelines in isolation is unlikely to be sufficient.

Secondly, while the guidelines do offer recommendations about how an Indigenous community member or non-Indigenous frontline health worker may support someone having suicidal thoughts, and what they may need to know to be able to do this, they don’t specify how Indigenous suicide gatekeeper training programs should be developed, packaged and integrated within broader community-based programs. In developing training programs based on these guidelines, it is important to consider the findings of two recent reviews of Indigenous suicide prevention programs that both strongly indicated how important it is that programs have a commitment to Indigenous leadership, community consultation, and the use of culturally appropriate frameworks for talking holistically about mental health and suicide (for example, the concept of social and emotional wellbeing) [12, 30]. The Aboriginal and Torres Strait Islander Suicide Prevention Evaluation Project led by the University of Western Australia found that Indigenous suicide prevention programs that were culturally appropriate and had a strong basis in community engagement and ownership from the outset were more likely to be effective [30]. A review of the literature on Aboriginal suicide prevention programs conducted by the Black Dog Institute in Sydney found that while there was a dearth of rigorous program evaluations, the results that were available indicated the importance of employing a ‘whole of community’ approach and focusing on connectedness, belongingness and cultural heritage [12]. They found that program longevity appeared to be linked to community ownership, with those programs still in operation after several years being those that started small, were wholly owned and run by the communities in which they were originated, and were connected to a broader suite of community developments. Additionally, they recognised that those Indigenous suicide programs that were wholly or partly Indigenous owned tended to employ creative methods of delivery, including art classes, dancing events, theatrical showcases, cultural camps and community activities, which may all be highly effective ways for some Indigenous communities to engage with the recommendations in these guidelines.

Thirdly, one common strategy of suicide gatekeeper-training programs, including those that would evolve from these guidelines, is to support people ‘at risk’ to link with mental health services. We must acknowledge that referring Aboriginal or Torres Strait Islander people to mental health services is neither unproblematic nor apolitical. There are important barriers that prevent formal mental health services from being an ideal source of care for Indigenous people. These have been documented in Australia, Canada the United States and elsewhere, for example: 1) the stigma and shame Indigenous people may experience when accessing formal mental health services; 2) experiences of discrimination and racism within the broader health system, which can in turn worsen psychological distress; 3) the provision of individualised care, rather than community- or family-based interventions, that diminishes the value that many Indigenous people place on interconnectedness; 4) a heavy reliance on individualised treatment options (predominantly pharmacological and psychological) that can be seen to de-contextualise experiences of suicidality in connection to structural issues like intergenerational trauma, racism, discrimination and disempowerment; 5) concerns that formal mental health services may not be provided in a way that is compatible with the holistic and strengths-based nature of the social and emotional wellbeing framing of Indigenous mental health; and 6) a lack of engagement with cultural and/or spiritual approaches to nurturing social and emotional wellbeing (for example, community gatherings, intergenerational transmission of knowledge and stories, dancing, healing ceremonies, and nature-based activities), which are largely distanced as being outside the bounds of evidence-based mental health care [12, 31–40]. Formal mental health services are a critical resource for Aboriginal and Torres Strait Islander people experiencing suicidal thoughts, however, the challenges are many for them to become culturally safe and appropriate sources of care. Indigenous suicide gatekeeper programs can acknowledge these shortcomings and work with communities to discuss and establish acceptable ways of accessing support and care from different sources, while advocating with mental health services around the need to develop holistic, flexible and culturally appropriate approaches.

### Limitations and future research

These guidelines have utilised the expertise of Indigenous suicide experts to offer recommendations as to how to support an Aboriginal or Torres Strait Islander who is experiencing suicidal thoughts or displaying suicidal behaviour. An important next step will be to conduct a trial to evaluate the outcomes of training programs that are based on these guidelines, in terms of their effect on participant knowledge, skills, intentions to assist and confidence in identifying and assisting individuals at risk of suicide. It will also be important to examine longer-term outcome measures, for example, assessing actual experiences of providing support against the guidelines and monitoring patterns of referrals to community supports and health services. Additionally, it is important to assess the perceived cultural appropriateness of these training programs for the participants.

Our panel was formed entirely of Aboriginal and Torres Strait Islander people with professional experience in Indigenous suicide prevention. Future research could consider also having a panel of people who identify as consumers of suicide prevention services or carers of people who are or have been suicidal, as such people would bring a different type of equally important expertise that would add great value to the re-development of these guidelines. However, given the high rate of suicide deaths in Aboriginal and Torres Strait Islander communities, it is not surprising that all of our professional panel members had personal exposure to suicidal thoughts and behaviour in either themselves, their families, their friends, or in their broader community network.

It must be kept in mind that the helping actions endorsed in the guidelines are based on expert opinion; these are the recommendations of experts in the absence of evidence from experimental studies about how best to provide mental health first aid to an Aboriginal or Torres Strait Islander person experiencing suicidal thoughts. Additionally, the use of these guidelines is recommended for use by mental health first aiders only. While the actions endorsed in these guidelines may be useful in different aspects of the Indigenous suicide prevention continuum, from preventing the onset of suicidal ideation itself to supporting the suicidal person in a professional setting, these are specific to the recommended support that can be provided by first aiders. These guidelines take into consideration the limitations in the first aiders’ support role, and guide the first aider on how to act within these. Nonetheless, these guidelines may be useful to those working on Indigenous suicide prevention outside the scope of the mental health first aid paradigm. Indeed, qualitative research should be undertaken to examine the perceived utility of these guidelines for those developing or implementing Indigenous suicide prevention programs, including suicide gatekeeper training courses, across Australia.

The majority of the suicide prevention literature is based on studies and reports that are not specific to Aboriginal and Torres Strait Islander peoples, or other Indigenous communities in other countries. Thus, the majority of the statements presented to our Aboriginal and Torres Strait Islander panel, for endorsement or otherwise, were generated from the mainstream suicide prevention literature, which does not necessarily embody the holistic and Indigenous-preferred concept of social and emotional well-being. This put a great onus on our expert panel to either suggest new culturally appropriate helping statements or to suggest re-wording of existing actions so that they were more culturally appropriate. This was a difficult task for panel members given they were already faced with reviewing a large number of helping statements.

Finally, only two panel members identified as Torres Strait Islander, which may affect the generalisability of the findings for Torres Strait Islander peoples.

## Conclusions

Through the Delphi process, the Aboriginal mental health first aid guidelines for supporting an Aboriginal or Torres Strait Islander person experiencing suicidal thoughts or displaying suicidal behaviour have been updated to ensure they are current and include the most recent and appropriate helping actions. This re-development has added depth to the previous version of the guidelines. These guidelines will now be made freely available for download on the MHFAA website, and will also be used to form the basis of an AMHFA Indigenous suicide prevention gatekeeper training course aimed at educating members of the public in providing first aid to an Aboriginal or Torres Strait Islander person who is experiencing suicidal thoughts.

## Additional file

Additional file 1: Items endorsed and rejected from the guidelines. The data supporting our findings is attached as Additional file 1, which contains the ratings for all the statements that were presented to the panel. (XLSX 31 kb).

## Abbreviations

AMHFA: Aboriginal Mental Health First Aid
MHFAA: Mental Health First Aid Australia
